# Pediatric emergency department visits during the COVID-19 pandemic: a large retrospective population-based study

**DOI:** 10.1186/s13052-021-01168-4

**Published:** 2021-11-04

**Authors:** Claudio Barbiellini Amidei, Alessandra Buja, Andrea Bardin, Filippo Bonaldi, Matteo Paganini, Mariagiovanna Manfredi, Andrea Favaro, Vincenzo Baldo, Mario Saia, Liviana Da Dalt

**Affiliations:** 1 Department of Cardiologic, Vascular and Thoracic Sciences and Public Health, University of Padova, Via Loredan, 18, 35131 Padova, Italy; 2 Department of Biomedical Sciences, University of Padova, Padova, Italy; 3 Emergency Department and Emergency Medical Service, “San Bassiano” Hospital, ULSS 7 “Pedemontana”, Bassano del Grappa, Italy; 4 Clinical Governance Unit, Azienda Zero, Padova, Italy

**Keywords:** Pandemic, Children, Emergency department, Access to healthcare services, COVID-19

## Abstract

**Background:**

COVID-19 pandemic has stretched healthcare system capacities worldwide and deterred people from seeking medical support at Emergency Departments (ED). Nevertheless, population-based studies examining the consequences on children are lacking.

**Methods:**

All ED visits from 2019 to 2020 in Veneto, Italy (4.9 million residents) were collected. Anonymized records of pediatric (≤14 years) ED visits included patient characteristics, arrival mode, triage code, clinical presentation, and discharge mode. Year-on-year variation of the main ED visit characteristics, and descriptive trends throughout the study period have been examined.

**Results:**

Overall, 425,875 ED presentations were collected, 279,481 in 2019, and 146,394 in 2020 (− 48%), with a peak (− 79%) in March–April (first pandemic wave), and a second peak (below − 60%) in November–December (second pandemic wave). Burn or trauma, and fever were the two most common clinical presentations. Visits for nonurgent conditions underwent the strongest reduction during both pandemic waves, while urgent conditions reduced less sharply. ED arrival by ambulance was more common in 2020 (4.5%) than 2019 (3.5%), with a higher proportion of red triage codes (0.5%, and 0.4% respectively), and hospitalizations following ED discharge (9.1%, and 5.9% respectively).

**Conclusion:**

Since the beginning of the COVID-19 pandemic, pediatric ED presentations underwent a steeper reduction than that observed for adults. Lockdown and fear of contagion in hospital-based services likely deterred parents from seeking medical support for their children. Given COVID-19 could become endemic, it is imperative that public health experts guarantee unhindered access to medical support for urgent, and less urgent health conditions, while minimizing infectious disease risks, to prevent children from suffering direct and indirect consequences of the pandemic.

**Supplementary Information:**

The online version contains supplementary material available at 10.1186/s13052-021-01168-4.

## Introduction

Since December 2019, 150 million people worldwide have been infected with SARS-CoV-2, and 3.15 million have died as of April 2021 [1]. Italy was hit particularly hard during the first, and second waves of the COVID-19 pandemic (in spring and autumn 2020). By the end of December 2020, over 2.1 million cases and 77,583 deaths had been recorded in the country [2]. Children and adolescents accounted for only about 1–2% of confirmed COVID-19 cases [3], without age differences in the infection rates [4].

After the first clusters of COVID-19 cases were detected in Italy in late February 2020 [5], the government implemented social distancing rules and imposed strict restrictions on individual mobility for the whole country starting from March 10^th^. Sixty million people were placed under lockdown in the attempt to counter the disease’s spread, and contain the burden, especially on hospital-based services and intensive care units [2]. The restrictions were gradually eased from May 2020 onwards, as the epidemic curve began to decline significantly. In October 2020, Italy was hit by a second wave of infections, prompting the government to reintroduce most of the restrictions adopted during the first wave. As the epidemic spread, intensive care units and hospitals dedicated to COVID-19 patients experienced a surge of admissions, while the use of other healthcare services decreased. In Italy, as in other hard-hit countries, the measures taken to cope with the pandemic led to a decline in the access rates to numerous healthcare services, including general practitioner and specialist visits, hospital admissions [6, 7], and emergency department (ED) visits [6, 8, 9]. Outpatient visits have recorded one of the strongest and most persistent drops since the start of the pandemic [10].

From a clinical perspective, children with SARS-CoV-2 infection are often asymptomatic or develop only a mild, shorter-lived illness compared to adults [11–14], but severe cases of pediatric COVID-19 have been described [15, 16].

A Spanish study reported a reduction in pediatric ED visits during the country’s first pandemic wave, although the percentage of hospitalizations following ED visits had doubled [17]. This finding is suggestive of a tendency for parents to refrain from presenting their children at the ED during the pandemic, in absence of severe health conditions [18–21]. However, extensive evaluations of the impact of the COVID-19 pandemic on ED visits among children are currently lacking.

The aim of the present study was to conduct a detailed analysis of ED visits during 2019 and 2020, examining a large pediatric population in the Veneto region, northeastern Italy. We compared pre- and post-pandemic trends in ED visits, focusing on different types of clinical presentations, and specific pediatric ED trajectories.

## Methods

### Context

In Italy, healthcare is made available to all citizens and residents through a mixed public-private system. The system is taxpayer-funded and administered on a regional basis, under regulations issued by the national Ministry of Health. The core principles of Italy’s National Healthcare Service (NHS) are universality and equity of care. EDs provide non-stop free access for medical urgencies and emergencies. In absence of urgent conditions, patients are charged for the ED visit unless they are then hospitalized, or admitted to the brief-stay intensive care unit.

The present study was conducted in the Veneto region, northeastern Italy, which has a resident population of 4.9 million, with 52 active EDs at the time of the study (46 public and 6 private). Healthcare facilities in this area are organized into a regional network comprising: a) 7 major “hub” hospitals (including 2 university hospitals) with highly-specialized services, located in the major cities; b) 24 medium-sized “spoke” hospitals, each serving an average population of 250,000; and c) 21 small local hospitals. The smaller hospitals provide basic first-aid services, while the larger hospitals manage more complex conditions, pediatric surgical procedures, neonatal intensive care, and there are dedicated pediatric intensive care units [22, 23]. Veneto was initially one of the regions in Italy most severely affected by the COVID-19 epidemic, but its early public health response—which included a thorough case-finding and contact-tracing system, as well as a substantial increase in intensive care unit capacity, helped to prevent any breakdown of the regional healthcare system [5, 12].

### Materials

Records of all children aged 14 or less arriving at any ED in Veneto during 2019 and 2020 were collected from a regional database where all ED visits are automatically recorded. The information available for each ED visit included: date of access, arrival mode, age, sex, clinical presentation, triage color code, and outcome. Three age brackets were chosen: less than 1 year, from 1 to 5 years, and from 6 to 14 years. Access mode was classified as walk-in, or by ambulance. Clinical presentations were grouped in one of the following 27 clinical categories: abdominal pain, acute neurological syndrome, allergic reactions, burn or trauma, chest pain, coma, dermatological symptoms, dyspnea, ear, nose and throat (ENT) disorders, fever, foreign object inhalation, forensic/legal medicine, gynecological disorders, hypertension, irritability, muscle pain, nephrological-urological disorders, nontraumatic hemorrhage, odontostomatological diseases, ophthalmological symptoms, other nervous system symptoms, other symptoms, poisoning, seizures, shock, social problems, tachycardia and palpitations. Triage color codes used to prioritize patients on arrival were: white tags for the mildest, not urgent conditions (lowest priority); green tags for mild conditions (low priority); yellow tags for urgent, potentially life-threatening conditions (high priority); and red tags for critical, life-threatening conditions (highest priority). Outcomes were classified as discharge at home, hospitalization, or death. Among all records, age was missing in 79 cases in 2019 and 3951 cases in 2020. These records were excluded from our analyses. Population data on confirmed cases of COVID-19, and patients hospitalized with symptoms probably attributable to the disease in Veneto region were retrieved from the database of the Italian Civil Protection Department, which monitors the coronavirus pandemic [13].

### Statistical analyses

Monthly ED visits in 2020 were plotted against the number of new daily confirmed SARS-CoV-2 infections, as well as the number of hospitalized patients with symptoms suggestive of COVID-19 in Veneto, in the same period.

We calculated the variation in the number of ED visits in 2019 and 2020, grouped by: sex, age bracket, month of the year, triage color code, and clinical presentation. The bimestrial trend of ED visits for the 10 most common clinical presentations (over the entire study period) was then calculated for 2019 and 2020. The year-on-year percentage variation in the number of ED visits per month (comparing the same month in 2019 and in 2020) was measured, overall and stratified by triage code. The trajectories of ED visits grouped by arrival mode, triage code (excluding white color codes), and outcome were calculated for each of the two years considered, and analyzed descriptively. All analyses were carried out with R statistical software and Microsoft Excel.

### Ethical statement

This study was conducted on data routinely collected by the healthcare services, using anonymized records. All analyses were performed on aggregated data. All data in Local Health Authority registries are recorded with the patient’s consent and, once fully anonymized, can be used for research purposes without further authorization (*Garante per la protezione dei dati personali,* Resolution n. 85, March 1^st^, 2012). This study complies with the Declaration of Helsinki and the Italian Decree n. 196/2003 on personal data protection.

## Results

**Fig. 1:**
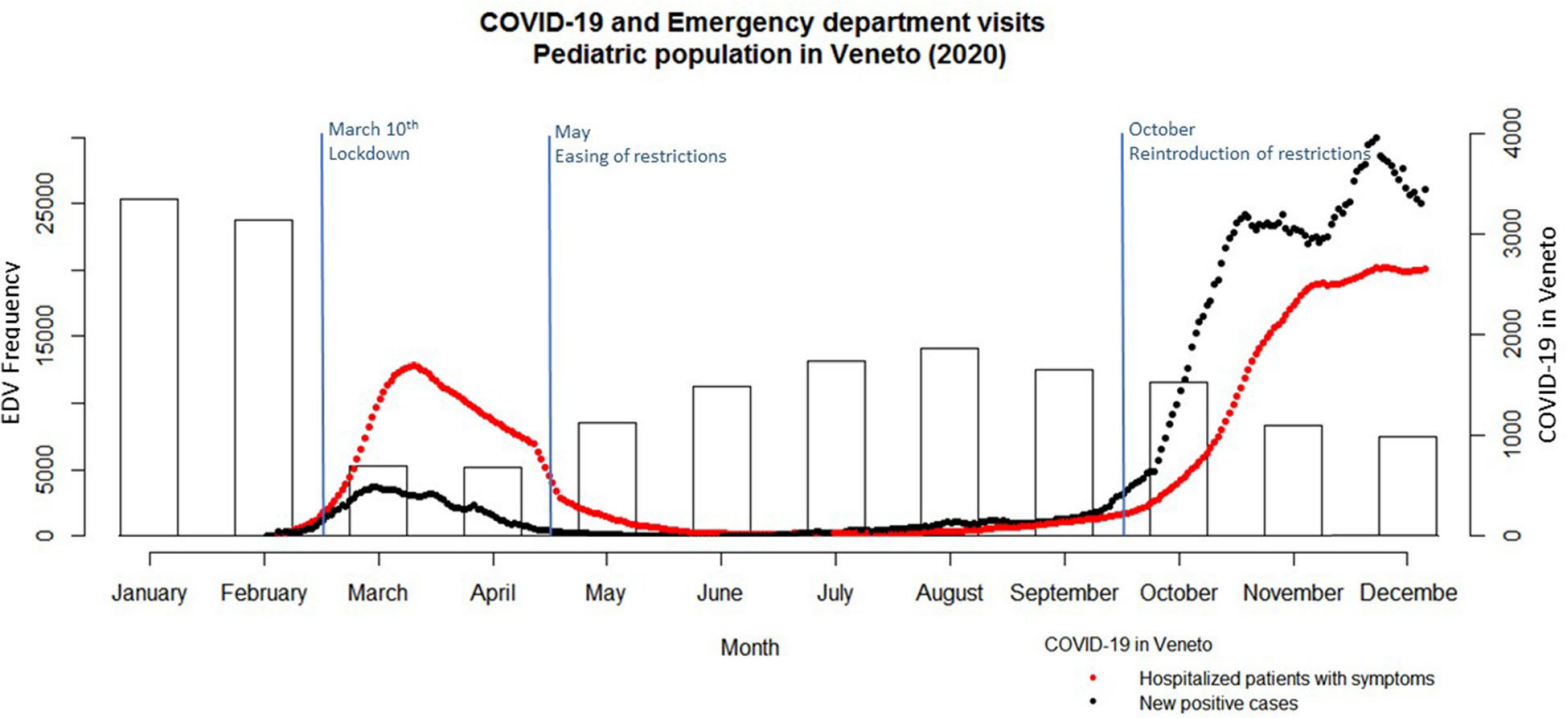
Pediatric emergency department visits (EDV) in Veneto (Italy) and the most significant governmental interventions, plotted against the number of hospitalized patients with COVID-19 symptoms, and daily incidence rate of SARS-CoV-2 infections, in 2020.* * y-axis 1 (left): frequency of pediatric EDVs; y-axis 2 (right): number of hospitalized patients with COVID-19 symptoms, and daily incidence rate of new SARS-CoV-2 infections

In 2019 there were 279,481 ED visits in Veneto of children aged 14 years or less. In 2020 the visits almost halved, falling to 146,394. Figure 1 shows the number of monthly ED visits in 2020 plotted against the new daily cases of SARS-CoV-2 infections and hospitalized patients with COVID-19 symptoms, in Veneto region. A sudden drop in ED visits is already apparent in March and April 2020, at the time of the first wave of the COVID-19 pandemic in Europe. The number of COVID-19 cases started to decrease from May 2020, followed by a gradual increase in the frequency of pediatric ED visits during the summer. The number of visits then dropped again during autumn, although less sharply, as the second pandemic wave hit.

Table 1 shows the differences in the characteristics of pediatric ED visits in 2019 and 2020. The proportion of male and female patients did not vary substantially, nor did the age, despite a very sharp decrease (− 51.2%) among 1- to 5-year-olds in 2020. In the month-by-month comparison, the largest drop occurred in March and April 2020 (− 78.7% and − 78.6%, respectively, compared with the same months in 2019). In both years, slightly more than half of all ED visits involved patients with non-urgent conditions (white triage color tags). There were slightly more cases tagged as yellow (urgent, potentially life-threatening conditions) in 2020 (10.3%) than in 2019 (8.2%), and the same was true for red triage codes (critical, life-threatening conditions), with 0.5% of all ED visits in 2020 as opposed to 0.4% in 2019.

**Table 1:** Pediatric emergency department visits in 2019 and 2020, grouped by sex, age, month, and triage color code

	2019		2020		Variation 2019–2020
	N	%	N	%	%
**Overall**	279,481		146,394		−47.6%
**Sex** ^**a**^					
Male	156,904	56.1%	83,461	57.0%	−46.8%
Female	122,576	43.9%	62,933	43.0%	−48.7%
**Age**					
0 years	34,548	12.4%	18,716	12.8%	− 45.8%
1–5 years	118,746	42.5%	57,942	39.6%	−51.2%
6–14 years	126,187	45.2%	69,736	47.6%	− 44.7%
**Month**					
January	24,973	8.9%	25,340	17.3%	1.5%
February	25,449	9.1%	23,747	16.2%	−6.7%
March	24,764	8.9%	5287	3.6%	−78.7%
April	24,235	8.7%	5180	3.5%	−78.6%
May	23,052	8.3%	8573	5.9%	− 62.8%
June	26,957	9.7%	11,236	7.7%	−58.3%
July	22,803	8.2%	13,157	9.0%	−42.3%
August	20,829	7.5%	14,062	9.6%	− 32.5%
September	18,626	6.7%	12,519	8.6%	−32.8%
October	21,875	7.8%	11,577	7.9%	−47.1%
November	21,271	7.6%	8273	5.7%	−61.1%
December	24,647	8.8%	7443	5.1%	− 69.8%
**Triage code**					
Red	1239	0.4%	749	0.5%	−39.5%
Yellow ^**b**^	23,031	8.2%	15,013	10.3%	−34.8%
Green	101,807	36.4%	50,994	34.8%	−49.9%
White	152,124	54.4%	78,755	53.8%	−48.2%
Not indicated ^**c**^	1280	0.5%	883	0.6%	−31.0%

^***a***^*Sex was missing in 1 record*

^***b***^*Orange triage color codes introduced in 2020 were merged with yellow codes for consistency across the study period*

^***c***^*Not indicated: includes patients arriving at the ED, but not being examined there (*e.g.*, administrative registration)*

**Fig. 2:**
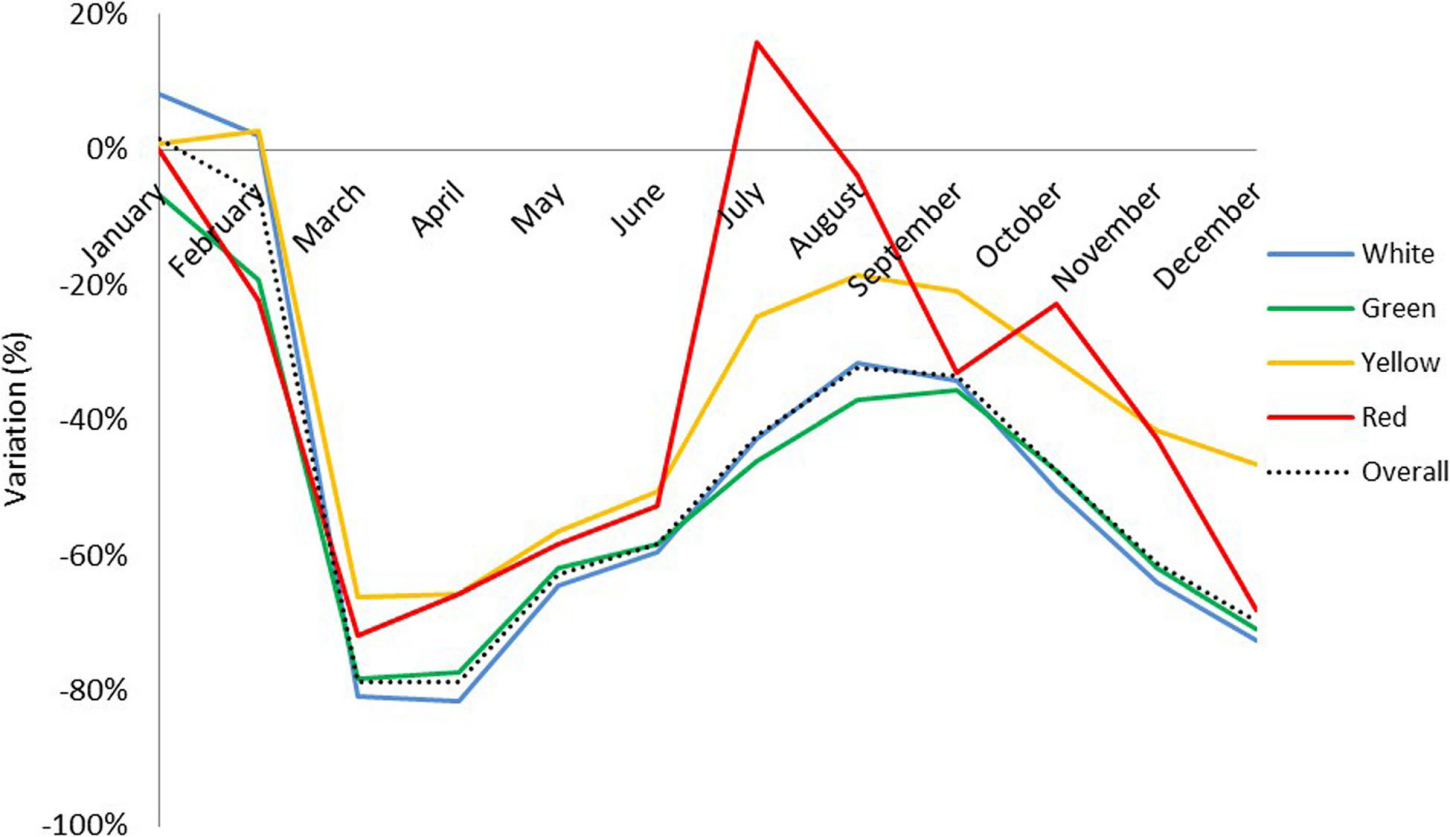
Monthly year-on-year variation of triage color codes assigned to children in emergency departments in 2019 and 2020. *Orange triage color codes introduced in 2020 were merged with yellow codes for consistency across the study period*

Figure 2 shows the trends in the month-by-month variation grouped by triage color code, between 2019 and 2020. There was a steep drop in ED visits in spring 2020 for all triage codes, although visits that were assigned red, or yellow triage tags (clinical urgencies, and emergencies) decreased slightly less than green and white tags (non-urgent, or mild conditions). Comparing the two years, there is a reduction in all ED visits, for all triage codes in 2020, except for January (+ 8.2 % for white tags, in pre-pandemic times), and July (+ 16% for red tags). When comparing 2019 with 2020 by triage code, the proportion of children with white, or green triage tags decreased more sharply (− 48.2% and − 49.9%, respectively) than those with a yellow or red tag (− 34.8% and − 39.5%, respectively).

Clinical presentation also varied significantly in 2019 and 2020. Burns and traumas were by far the most common clinical presentation in both years, accounting respectively for 27.9% and 32.1% of all pediatric ED visits - although the number of visits for burns or traumas decreased by 39.8%. As seen for ED visits overall, there was a substantial reduction from 2019 to 2020 in the number of ED visits for almost all clinical presentations considered. The most marked reductions concerned: irritability (− 71.0%), muscle pain (− 70.7%), forensic/legal medicine (− 64.2%), other nervous system symptoms (− 61.0%), and seizures (− 60.7%). ED visits for fever and dyspnea also decreased substantially in 2020 (− 47.7% and − 48.3%, respectively), but they continued to account for the same proportions of clinical presentations (4.5% and 14.1%, respectively) in both years, as shown in Table 2.

**Table 2:** Variation in pediatric clinical presentations at emergency departments in 2019 and 2020, in Veneto region, Italy

Clinical presentation	2019		2020		Variation 2019–2020
	N	%	N	%	%
Abdominal pain	25,764	9.2%	10,414	7.1%	− 59.6%
Acute neurological syndrome	647	0.2%	494	0.3%	−23.6%
Allergic reactions	1953	0.7%	865	0.6%	−55.7%
Burn or trauma	77,799	27.9%	46,826	32.1%	−39.8%
Chest pain	1714	0.6%	898	0.6%	−47.6%
Coma	512	0.2%	367	0.3%	−28.3%
Dermatological symptoms	9501	3.4%	3975	2.7%	−58.2%
Dyspnea	12,559	4.5%	6495	4.5%	−48.3%
ENT disorders ^a^	12,073	4.3%	5405	3.7%	−55.2%
Fever	39,382	14.1%	20,609	14.1%	−47.7%
Foreign object inhalation	674	0.2%	288	0.2%	−57.3%
Forensic/legal medicine	215	0.1%	77	0.1%	−64.2%
Gynecological disorders	247	0.1%	154	0.1%	− 37.7%
Hypertension	7	0.0%	11	0.0%	57.1%
Irritability	1655	0.6%	480	0.3%	−71.0%
Muscle pain	1429	0.5%	419	0.3%	−70.7%
Nephrological-urological disorders	2718	1.0%	1691	1.2%	−37.8%
Nontraumatic hemorrhage	774	0.3%	488	0.3%	−37.0%
Odontostomatological diseases	1061	0.4%	547	0.4%	−48.4%
Ophthalmological symptoms	4924	1.8%	2456	1.7%	−50.1%
Other nervous system symptoms	7855	2.8%	3060	2.1%	−61.0%
Other symptoms	73,461	26.3%	38,679	26.5%	−47.3%
Poisoning	616	0.2%	477	0.3%	−22.6%
Seizures	509	0.2%	200	0.1%	−60.7%
Shock	220	0.1%	118	0.1%	−46.4%
Social problems	108	0.0%	83	0.1%	−23.1%
Tachycardia and palpitations	753	0.3%	388	0.3%	−48.5%
**Overall** ^**b**^	**279,130**	**100.0%**	**145,964**	**100.0%**	**−47.7%**

^*a*^*ENT: Ear, nose and throat*

^*b*^*Records with missing clinical presentations have been excluded from the analyses (N = 781)*

**Fig. 3:**
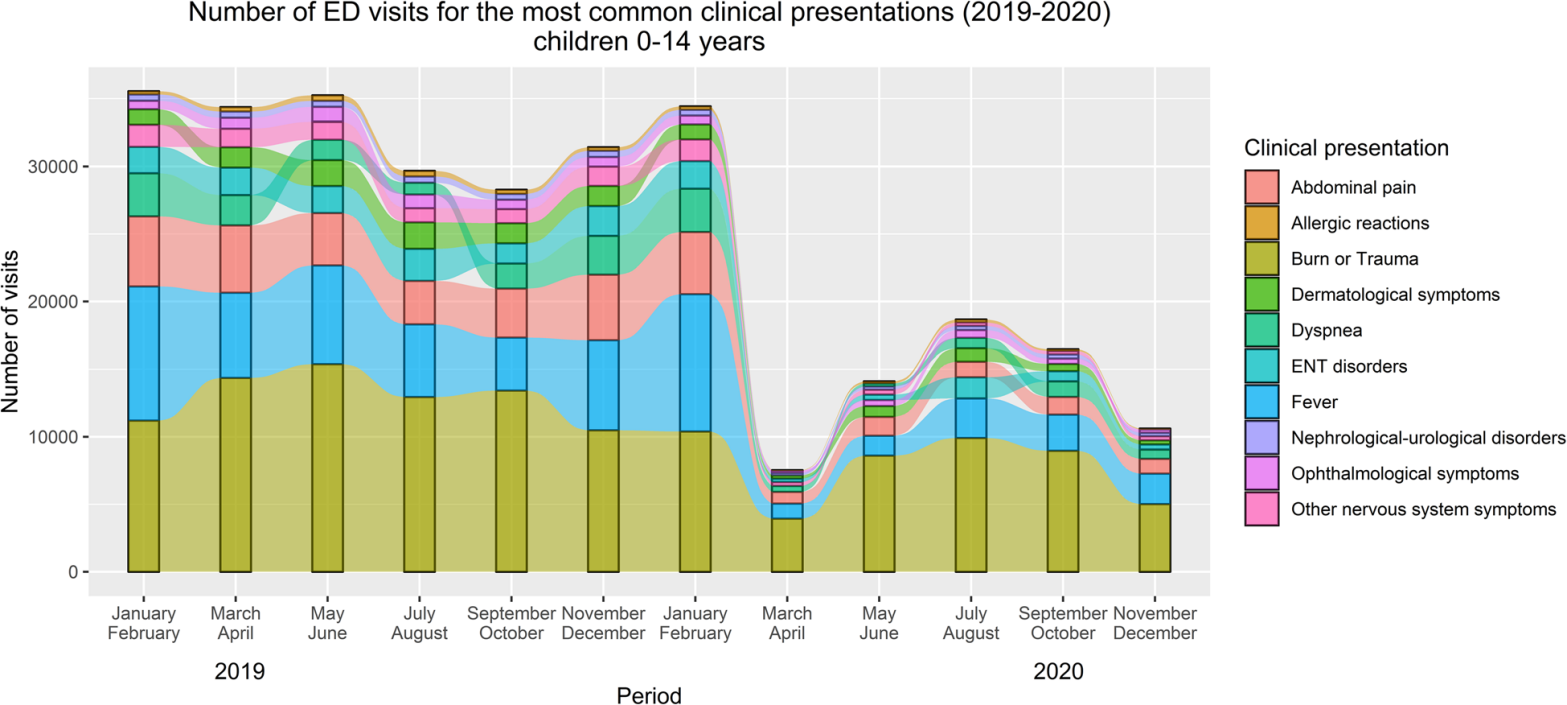
Bimestrial trend of the most common clinical presentations among children at emergency departments in 2019 and 2020 (N = 296,324 visits). Less frequent, unspecified and missing clinical presentations were excluded from this analysis (N = 129,551). *ENT disorders: Ear, Nose, and Throat disorders

Figure 3 shows the bimestrial trends of the ten most common clinical presentations over the two years**.** Once again, the marked drop in the number of ED visits starting in March 2020 (and continuing throughout the year) concerned all these presentations. Burns or traumas remained the most common clinical presentation throughout the entire study period, followed by fever. Abdominal pain was the third most common presentation, except in the months of July and August 2020, when ear, nose and throat disorders became the third most frequent cause for pediatric ED visits. Overall, there were no major changes in the proportion of each clinical presentation, as the sharp decline in ED visits affected them relatively homogenously. A subgroup analysis by age was also performed (Additional file 1), suggesting there are age-specific differences in the most frequent pediatric clinical presentations, although the overall impact of the pandemic, seems to have similarly affected ED visits across all age groups.

**Fig. 4:**
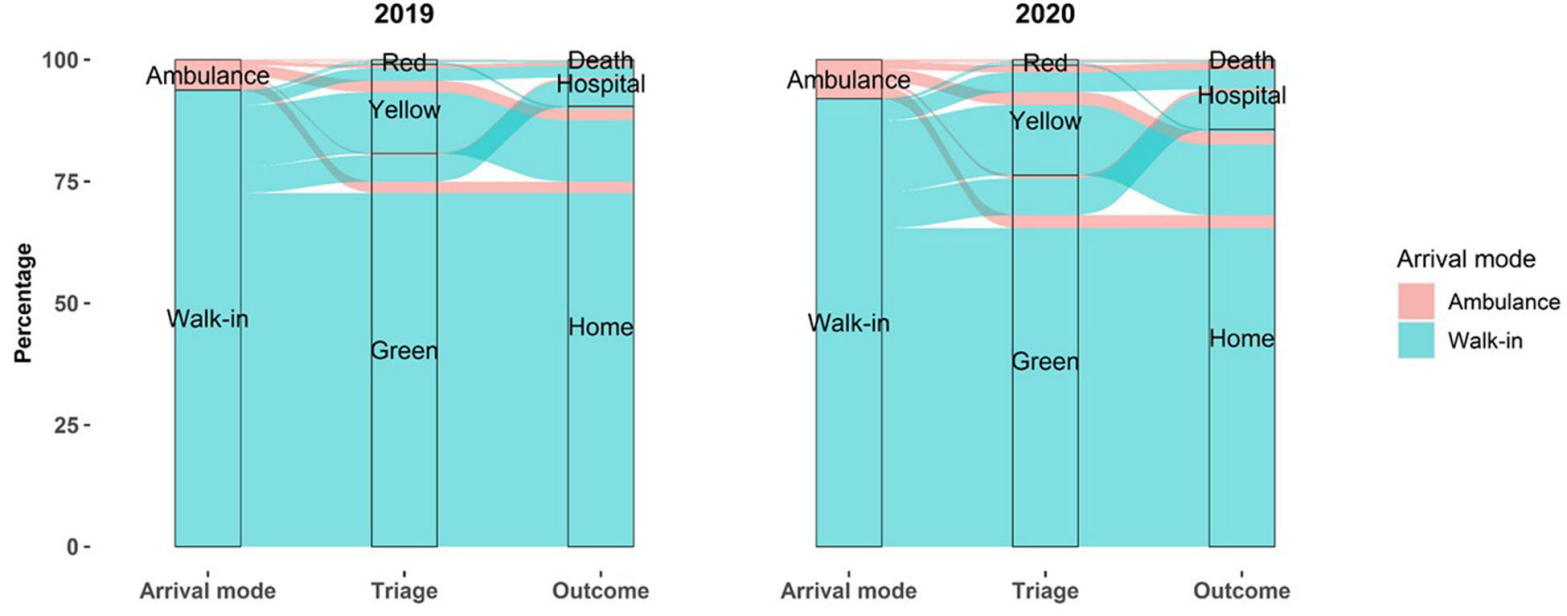
Trajectories of pediatric emergency department visits in 2019 and 2020 grouped by arrival mode, triage code, and outcome. *All trajectories with missing data on arrival mode, triage color code, or discharge mode were excluded from the analyses (N = 4603). Visits with a white triage code (N = 232,010) were excluded to better appreciate the most relevant changes among the less frequent triage codes (*i.e.*, red, yellow, and green tags). Orange triage color codes introduced in 2020 were merged with yellow codes for consistency across the study period*

Figure 4 shows the trajectories of pediatric ED visits in 2019 and 2020 grouped by arrival mode, triage code (excluding white tags), and outcome. Arrival by ambulance rose in 2020 from 3.5 to 4.5%, and the proportion of yellow, and red triage codes rose from 8.7 to 10.8% (data not shown). Outcomes such as death were extremely rare in both years (20 cases in 2019, and 15 in 2020), but the rate of hospital admissions following ED visits rose from 5.9% in 2019 to 9.1% in 2020, and the proportion of patients with home discharge decreased from 94.1 to 90.9%.

## Discussion

To the best of our knowledge, few studies have examined in detail the effect of the COVID-19 pandemic on the trends of pediatric ED visits over two years. Many papers focused exclusively on ED admissions under lockdown, and very few studies were population-based [24–30].

The present study found a significant decrease in the number of pediatric ED visits in Veneto, in 2020 compared to the previous year. These findings suggest that the measures taken by the central government to contain the pandemic had a profound impact on the use of EDs, as well as on other healthcare services. They led to a sharp drop (− 48%) in all medical consultations compared with 2019, especially during the lockdown, when they decreased by 79% compared  to the same months of the previous year, with a similar magnitude to that reported previously [30]. While the reduction in ED visits was more pronounced during the first and second waves of the pandemic, even with the loosening of restrictions during summer, they did not return to pre-pandemic levels. There could be various reasons for the sharp reduction during the pandemic, in addition to the authorities urging people to avoid using hospital-based services unless it was strictly necessary. For instance, among children and adolescents, limited individual mobility lowered the risk of traumatic injuries, and social distancing reduced the chances of contracting other communicable diseases. It is an issue of concern, however, that parents might have avoided seeking hospital-based services for fear of contagion. This impression is supported by the greater proportion of urgencies and emergencies in 2020, indicating a general tendency of children to be presented at the ED for more severe conditions, as observed by a previous Italian study [30]. This is further confirmed by the 9.1% of pediatric patients arriving at the ED in 2020 being hospitalized, with a sharp increase compared to the 5.9% recorded in 2019. A similar phenomenon had already been observed in 2003, when the fear of contracting SARS from patients, and healthcare workers, had deterred people from seeking care [31]. Now that the epidemic curve has improved, large numbers of citizens, including adolescents, have been vaccinated against SARS-CoV-2, and restrictions are gradually being lifted, it will be very important to see whether ED visits will remain lower than those seen before the COVID-19 pandemic. It is imperative for public health services to ensure that urgent medical care needs continue to be met. Monitoring health parameters in children following such an unprecedented reduction in ED visits, might shed light on its possible consequences. It is particularly worth focusing on the nonurgent conditions that were reportedly responsible for overcrowding at the EDs in pre-COVID-19 times. Children under 14 years seem to be amongst the least vulnerable to COVID-19, with only 176 hospitalizations in Veneto up to April 2021 (and infants up to 12 months accounted for 56% of these hospitalizations). Future studies should therefore try to clarify whether the persistent reduction in pediatric ED visits has paved the way for a more appropriate use of this acute healthcare service, or whether children have been suffering, as an indirect consequence of COVID-19, from inadequate medical support [25, 32]. If no detrimental consequences were to be found, the pandemic could provide a unique opportunity to shift the center of pediatric emergency healthcare support to an integrated network of primary care pediatricians. This would allow to relieve hospital emergency departments from overcrowding for nonurgent conditions, and ensure more efficient support for patients presenting with severe conditions.

Our findings are consistent with previous literature on COVID-19 in other Italian regions. Several studies, in fact, reported a decline in all ED visits during the first lockdown that ranged from 62 to 81%, similarly to the rates found in the present study [25–27, 30]. The reduction seems to have affected the whole pediatric population to the same degree, without sex, or age differences [33].

In a cross-sectional study conducted on 27 pediatric EDs in the USA from mid-March to the end of August 2020, a decline in consultations could be seen even before the surge in SARS-CoV-2 cases. Interestingly, the study showed that the greatest decrease concerned visits for respiratory disorders. This could be explained by the widespread use of personal protective equipment (especially N95 respirators, and surgical masks) that led to a marked reduction in the circulation of all sorts of viruses, most of which had lower effective reproduction numbers (Rt) than SARS-CoV-2. The very low rates of flu observed during the flu season of 2019 and 2020 [34] are also suggestive of a possible effect played by personal protective equipment, combined with social distancing. The air filtering effect of personal protective equipment would also have reduced exposure to air pollution and pollens, ultimately leading to lower rates of respiratory symptoms (e.g. dyspnea, bronchitis, upper airway symptoms) [24]. Restricting social contacts, promoting intensive use of personal protective equipment, and potentiating hygiene measures (such as frequent hand washing) could also help explain the reduction in ED visits for clinical presentations relating to infectious diseases. Burns or traumas showed a less marked decline in our data (− 40%) compared to previous studies, probably linked to an increase in the number of domestic accidents [24, 35]. Similar trends to those found in Veneto region, were reported in two retrospective reviews concerning two tertiary pediatric hospitals in Singapore, which compared the first 6 months of 2020, with the same period in 2019 [28, 29]. The common findings of studies conducted in diverse populations suggest these phenomena might have similar effects in different settings.

The present work has several strengths, in particular a large population with a broad set of detailed information regarding each ED visit. Given our country’s national health service, the data discussed here are not at risk of bias, because almost all medical emergencies are managed by public hospitals, or publicly-funded private hospitals (that were included in our analyses). This enabled us to perform stratified analyses to shed light on specific phenomena that could have differentially affected particular subgroups during the COVID-19 pandemic. By representing trajectories (i.e. arrival mode, triage code, and outcome), we were able to compare ED paths before and after the pandemic. Limitations of this study include the lack of detailed information on comorbidities of children accessing the EDs, which prevented us from considering further clinical aspects, other than the type of clinical presentation on arrival at the ED. Certain clinical presentations underwent minor changes during the study period, but they were grouped to ensure full comparability. No data were available for children infected with SARS-CoV-2, so we could not examine the direct effects of the virus on pediatric ED presentations, in regards to arrival mode, triage urgency color tags, or outcome.

**In conclusion**, COVID-19 had a very strong impact in reducing the numbers of pediatric ED visits in Veneto region in 2020, and this was especially evident during the first lockdown in spring. The decline in ED consultations was especially marked for nonurgent medical conditions (white, and green triage tags), suggesting children with these conditions were less likely to be brought to an ED. The numbers of ED visits dropped steeply for all clinical presentations, but within each year, their ratio remained relatively stable, suggesting the absence of any differential effect of the pandemic on specific pediatric conditions.

The COVID-19 pandemic has prompted unprecedented changes in access to healthcare services that deserve to be examined in detail. Policy makers should monitor the long-term effects of the pandemic on children, and tailor policies to maximize the efficacy of healthcare services in countering the spread of infectious diseases, without negatively affecting the quality of acute healthcare support. In a world where SARS-CoV-2 variants could become endemic, or new viruses could develop pandemic characteristics, it is imperative to ensure that people in general, but children especially, be constantly guaranteed full medical support in case of medical urgencies or emergencies, in the safest possible environment.

## Supplementary Information

**Additional file 1.** Trends in emergency department visits stratified by age group (0, 1-5, and 6-14 years).

## Data Availability

The data supporting the findings of this study are held by the Veneto Epidemiological Registry and were used under license for the present work, but they are not publicly available. These data are nonetheless available from Alessandra Buja (alessandra.buja@unidp.it) on reasonable request, and subject to permission being obtained from the Veneto Epidemiological Registry (Veneto Regional Authority).
