# Supplementary material for: Pediatric emergency department visits during the COVID-19 pandemic: a large retrospective population-based study
Source: Ital J Pediatr. 2021 Nov 4;47:218. doi: 10.1186/s13052-021-01168-4 (PMC8567132; doi:10.1186/s13052-021-01168-4)
Supplement: Supplementary file 1 — Additional file 1. Trends in emergency department visits stratified by age group (0, 1-5, and 6-14 years). [file 13052_2021_1168_MOESM1_ESM.docx]

**Supplementary material**

**Trends in emergency department visits stratified by age group (0, 1-5, and 6-14 years)**

**Supplementary Figure 1.** Bimestrial trends of the most common emergency department visits among infants (0 years) in 2019 and 2020 in the Veneto region (N = 31,454 visits).


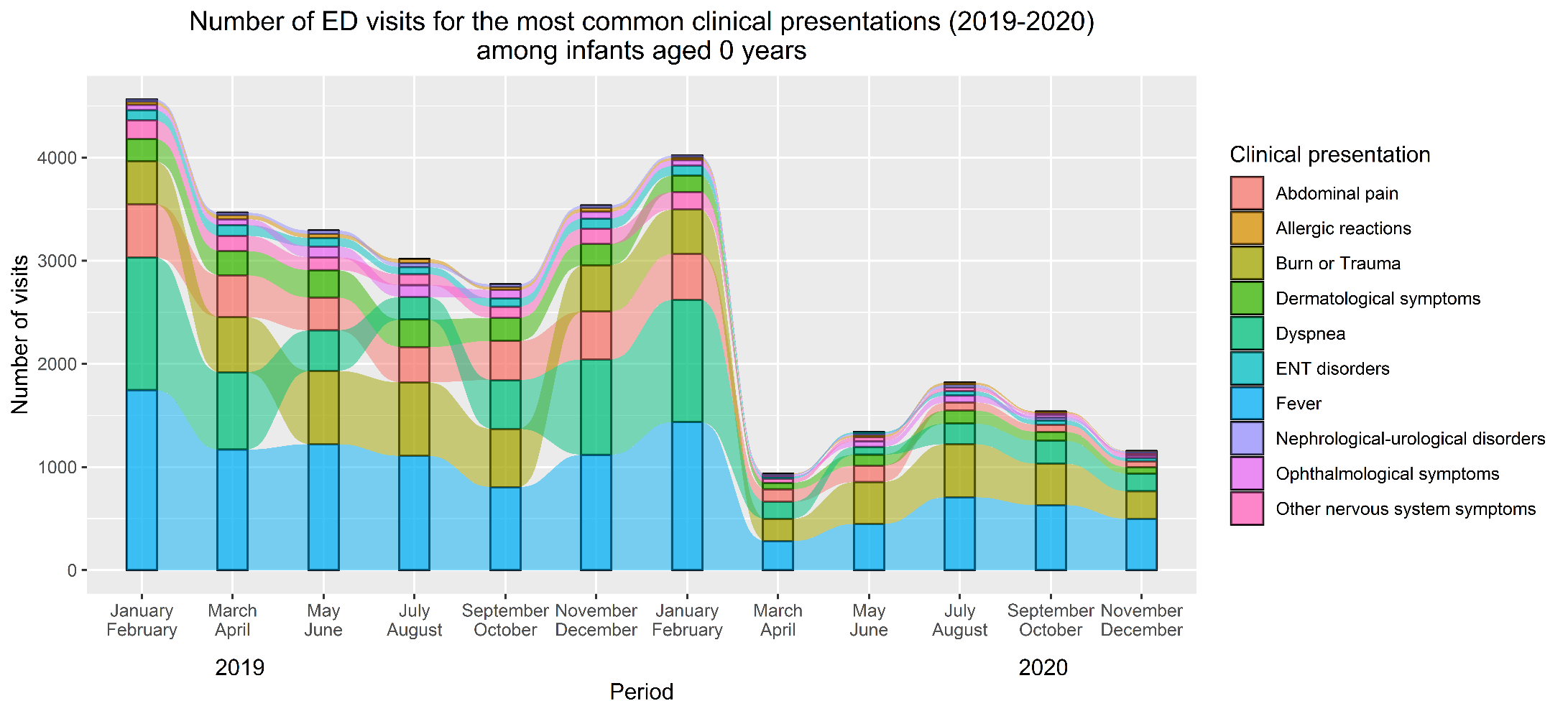


** All visits without a specific clinical presentation or with less frequent clinical presentations have been excluded from the analyses (N = 21,810).*

**Supplementary Figure 2.** Bimestrial trends of the most common emergency department visits among children aged 1 to 5 years in 2019 and 2020 in the Veneto region (N = 124,543 visits).


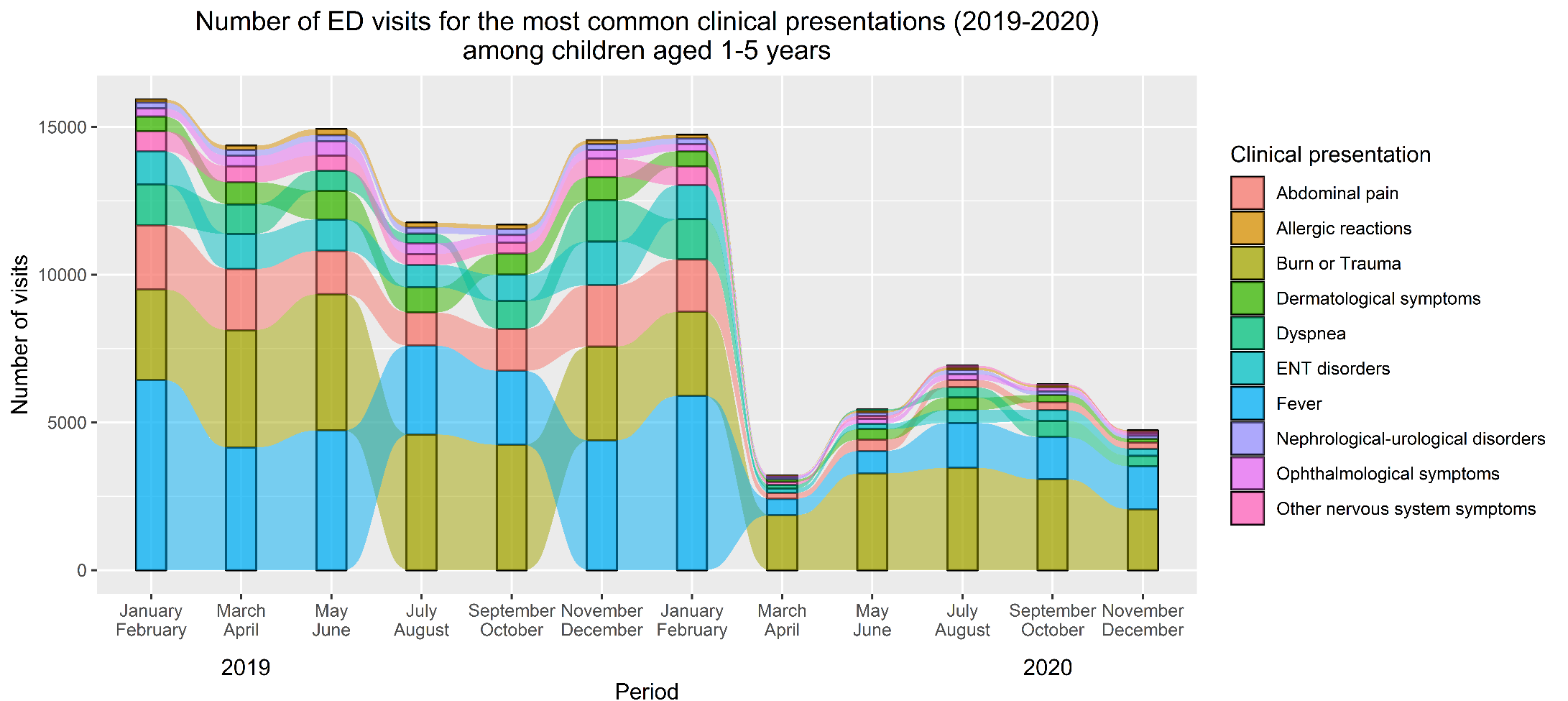


** All visits without a specific clinical presentation or with less frequent clinical presentations have been excluded from the analyses (N = 52,145).*

**Supplementary Figure 3.** Bimestrial trends of the most common emergency department visits among children aged 6 to 14 years in 2019 and 2020 in the Veneto region (N = 140,327 visits).


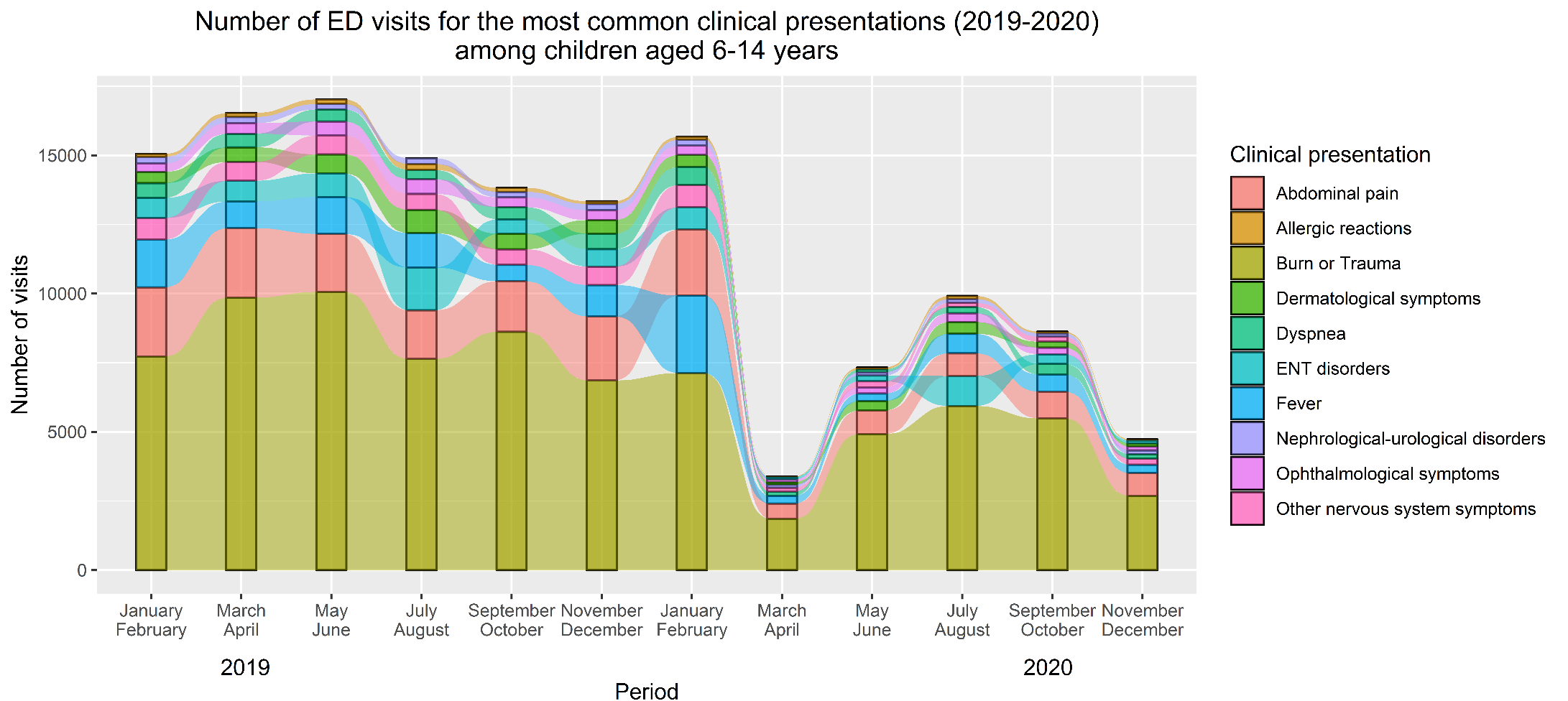


** All visits without a specific clinical presentation or with less frequent clinical presentations have been excluded from the analyses (N = 55,596).*
